# Health Disparity Still Exists in an Economically Well-Developed Society in Asia

**DOI:** 10.1371/journal.pone.0130424

**Published:** 2015-06-22

**Authors:** Albert Lee, Hoi-wai Chua, Mariana Chan, Patrick W. L. Leung, Jasmine W. S. Wong, Antonio A. T. Chuh

**Affiliations:** 1 Centre for Health Education and Health Promotion, The Chinese University of Hong Kong, Shatin, Hong Kong; 2 Hong Kong Council for Social Services, Wanchai, Hong Kong; 3 Department of Psychology, The Chinese University of Hong Kong, Shatin, Hong Kong; 4 JC School of Public Health and Primary Care, The Chinese University of Hong Kong, Shatin, Hong Kong; Old Dominion University, UNITED STATES

## Abstract

**Background:**

The socioeconomic inequalities in child health continue to widen despite improved economy.

**Objective:**

To investigate the correlation between socio-economic factors and health risk behaviors and psychosocial well-being of children in Hong Kong.

**Hypothesis:**

The null hypothesis is that for this particular developed region, there exists little or no correlation between social-economic factors and health risk behaviors and psychosocial well-being of children.

**Design:**

Cross sectional territory wide survey.

**Participants:**

Caregivers of 7,000 children in kindergartens in Hong Kong.

**Measuring tools:**

Youth Risk Behavior Surveillance questionnaire, health-related knowledge and hygienic practice questionnaire, and Children Behavior Checklist (CBCL).

**Results:**

Children were less likely to have somatic complaints and anxiety/depression as reflected by CBCL scores coming from families of higher income, not being recipients of social assistance, with fathers in employment, and with higher parental education. Children with only mother or father as caretakers had lower odds ratios (ORs) 0.71 (95% CI 0.58-0.89) and 0.53 (95% CI 0.33-0.84) respectively to have the habit of eating breakfast, whilst parental education at post-secondary level and higher family income had higher ORs 1.91 (95% CI 1.31-2.78), and 1.63 (95% CI 1.11-2.39). Fathers unemployed, relatives as main caretakers and living in districts with low median household inome incurred higher ORs, as 1.46 (95% CI 1.10-1.94),1.52 (95% CI 1.27-1.83) and 1.17 (95% CI 1.02-1.34) respectively, of watching television over two hours daily, whilst children with parental education at secondary level or above incurred lower OR 0.33 (95% CI 0.24-0.45). Children with parental education at post-secondary level and higher family income had lower ORs of 0.32 (95% CI 0.48-0.97) and 0.52 (95% CI 0.34-0.79) respectively, with regard to exposing to passive smoking, and reversed for those living in districts with lower median household income, lower family income and recipient of CSSA with ORs 1.24 (95% CI 1.06-1.44) and 1.6 (95% CI 1.09-2.37) respectively.

**Conclusion:**

Null hypothesis was not supported. A strong gradient was still found to exist among different socio-economic groups for various health-related behaviors in developed society like Hong Kong.

## Background

Socioeconomic inequalities in childhood morbidity and mortality are well documented worldwide [1]. In order to close the gaps of health inequity, the Commission on Social Determinants of Health examined the inequitable access to health and education as well as the factors constituting the social determinants of health [2–3]. The Commission emphasized the importance of early life investment in health by minimizing the risks of obesity, malnutrition, mental health and non-communicable diseases and enhancement of physical and cognitive developments [4]. The Commission also advocated routine monitoring systems for health equity and the social determinants of health locally, nationally and globally, with the aim of identifying and modifying health risk behaviors established early on in life [3, 5, 6]. In 2011, the United Nation Declaration of the General Assembly on the Prevention and Control of non-communicable diseases called for action to reduce the burden of those diseases [7].

Children in higher economic quintiles have been shown to be at lower risk of illnesses such as diarrhea and respiratory diseases after controlling environmental exposures and biological susceptibilities [8]. The health of adults is also determined by the physical constitutions during childhood [9], and poorer health and social outcomes were reported among children in families with low-incomes and recipients of social assistance [10]. Asymmetric economic growth, unplanned urbanization, marked environmental change, unequal improvements in daily living conditions, and the unequal distribution and access to quality health care have contributed to health inequities in the Asia Pacific region [11]. Although we have observed impressive improvements in overall indicators of health globally especially in Asia Pacific region over the last few decades, health inequalities continue to widen within and between countries and regions [12, 13, 14]. The ongoing monitoring of health inequities and the systematic evaluation of societal changes and their impact on health inequities would provide further evidence to address the geo-political and socio-economic diversity [11]. Although Putnam initially stated that health dependent factors such as diet and lifestyle were beyond the control of government, his subsequent work on the consequences of social capital revealed the importance of social connectedness to health and well-being [15]. Social capital has also been shown to have broader implications in the management of chronic diseases such as diabetes mellitus [16]. The effect of social capital on mortality was lessened when health related behaviors and material circumstances were included [17]. When socio-economic indicators at individual levels were controlled, no reduction in the risk of psychological distress was observed for each unit increase in community participation. This suggests the moderate effects of modifying socio-economic factors at individual levels [18]. Recent Lancet paper has reported the socio-economic inequalities in adolescent health from 2002–2010 among 34 European and North American countries, and these trends coincide with unequal distribution of income between rich and poor people [19]. In 2013, The Institute of Medicine, USA initiated ‘Forum on Investing in Children Globally’ to explore existing, new and innovative research from around the world and translate into sound and strategic investments in policies and practices that will make a difference in the lives of children [20]. Forum activities include public workshops and in April 2014 workshop, intersections across health, education, nutrition, living conditions, and social protection were explored and highlighted in the summary report of ‘Cost of Inaction on Investing in Children Globally [21]. It is therefore still important to continue examining the relationships between child health-related behaviors and socio-economic status in order to enlighten the policy approaches to promote health equity in children.

Investigations in the late 1990s have established the association of urbanization with greater deterioration in infant and child mortalities [22] and mental health problems [23]. Such known facts of impacts of socio-economic status and urbanization on child health in certain countries makes it important to study whether similar pattern applies to a highly urbanized region like Hong Kong, with a population density of 6,457 people/km^2^ (7.071 million in mid 2011 in 1,096 km^2^), high GDP per capita of US$ 34,457 (UK $38,188) [24], and widening of Gini coefficient from 0.525 in 2001 to 0.533 in 2006 and 0.537 in 2011 with 9^th^ and 10^th^ decile group of household income (highest income groups) dominating 56% of total income in Hong Kong [25–26]. Hong Kong has the highest Gini coefficient of household income among developed countries in Asia Pacific region such as Singapore (0.478 in 2008 and 0.463 in 2013), Japan (0.376 in 2008), South Korea (0.314 in 2009 and 0.311 in 2011) and Australia (0.305 in 2006 and 0.303 in 2008) [26]. All those countries except Australia had higher Gini coefficient compared with European Union (estimated 0.306 in 2012) and also Norway (0.25 in 2008) [26]. Hong Kong is experiencing rising trend while others are decreasing.

Disparity in income is more marked in Hong Kong. The objective of this study is to investigate the correlation between socio-economic factors and health risk behaviors and psychosocial well-being of children in Hong Kong, a developed region in China. The null hypothesis is that for Hong Kong, there exists little or no correlation between social-economic factors and health risk behaviors and the psychosocial well-being of children.

## Methodology

Our design is a cross sectional territory wide survey targeted at 7,000 children in kindergartens in Hong Kong.

### Ethical approval

The study was approved by The Chinese University of Hong Kong Survey and Behavior Research Ethics Committee. Letters were sent to parents of the participating kindergartens requesting them to consent to participate. All parents/guardians were provided written informed consent. The completed questionnaires were returned to the kindergartens, and then collected by the investigators. Only investigators were allowed access the anonymous questionnaires to ensure confidentiality.

### Measuring socio-economic factors related to Poverty

There is no universally accepted definition of poverty. The income eligibility for Comprehensive Social Security Allowance (CSSA) is commonly regarded as a poverty line. Relative poverty is a widely used concept in Hong Kong. The Commission of Poverty of Hong Kong Government adopted the concept of ‘relative poverty’ after taking reference from international experience and views from academics, non-government organisations (NGOs) and views of public to set the main poverty line at 50% off median household income (HK$10,000 monthly or US$1,250 for 2006) [27].

The socio-economic factors to be included for this study are education level of parents, the monthly income of families, the unemployment of parent, the recipient of CSSA, other subsidies (tuition fee/textbook subsidy/travel allowance), median monthly house hold income of the district where kindergartens were located (stratified in three categories: ≤HK$15,000, HK16,000 to below HK$20,000 and ≥ HK$20,000) reflecting the neighourhood where the children were living as children tended to attend kindergartens in their own catchment areas, status of school (whether it is supported by major School Sponsoring Body such as religious bodies, Catholic Diocese, Anglican Church, or large NGOs with long history of establishment) reflecting the education quality of kindergarten as kindergartens operated by major School Sponsoring Bodies would have better resources with regards to education supporting services), and the newly arrival status of parent(s) (less than seven years in Hong Kong, as residing in Hong Kong for seven consecutive years is the requirement for the right of abode in Hong Kong).

### Measuring tools

A cross-sectional survey on Youth Risk Behavior Survey based on self-administered questionnaires by parents was conducted by the Centre for Health Education and Health Promotion of the Chinese University of Hong Kong (CUHK) during the academic year of 2005/06 on kindergarten students. Parts of the questionnaire were adopted from the Youth Risk Behavior Survey (YRBS) by the USA Centre for Disease Control which had been used by this center to assess the health of children since 1999 [28]. Time spent on television was included in YRBS as this would lead to physical inactivity particularly in this region and physical inactivity has been shown as risk factor for cardiovascular disease [29]. Another part of the questionnaire testing health-related knowledge and hygienic practice was devised by CUHK research team which had undergone face and content validations [30]. The Children Behavior Checklist (CBCL), a parent-informant rating scale with parallel versions for school-aged children and preschoolers, were used to measure their psychological well-being covering three domains, namely anxiety/depression, somatic complaints, and aggressive behavior [31, 32]. These two forms share a large number of common attributes, and examine similar psychopathological constructs. The Chinese versions of these forms had their validity and applicability investigated for Chinese children [32, 33].

### Data collection and Participants

In this cross-sectional study, the health behaviors and mental health status were evaluated by parent-completed questionnaire. The participating kindergartens were 78 kindergartens when they first joined the Health Promoting Kindergarten Scheme initiated by CUHK in 2005. The scheme was open to all kindergartens in Hong Kong (around 1,000 during that period) so the participating kindergartens located in different geographic clusters of Hong Kong. The primary caregivers of kindergarten children were recruited. Children with special learning needs and non-Chinese were not included as the measuring tools were designed for caregivers of Chinese children. 7,057 questionnaires were completed by the primary caregivers, mostly parents (96.6%) with response rate over 90%.

### Statistical Analyses

Data on different variables related to health were tabulated as percentage and the CBCL for the three domains were presented as scores. Where applicable, Chi-square tests of independence were utilized to analyze the differences of health behaviors of children amongst different socio-economic groups as mentioned under the section on socio-economic factors at the 5% levels of significance. T-tests were used to analyze the differences of CBCL scores between different socio-economic groups. All those factors were entered as independent variables for stepwise multivariate analyses to identify the significant independent variable(s) reflecting socio-economic status correlated with various health behaviours or health status.

## Results

The analyses were based on 7,057 main caregivers of kindergarten children returning the questionnaires with valid data. The characteristics of the respondents are described in comparison with Hong Kong population in S1 Appendix.

S2 Appendix describes the prevalence of health and hygiene behaviors. Hygienic practices were generally found to be satisfactory (96% washing their hands before meals, except oral hygiene for which only 32% brushed their teeth after each meal). Over half had sub-optimal intake of fresh fruits (43% consumed less than one portion daily), vegetables (only 34% consumed at least one bowl of vegetable daily) and water (only 16% had at least five glasses daily). The level of physical activities was extremely low (only 16.5% had 60 minutes of physical activities at moderate intensity or above for at least one day per week). Nearly half (45%) were spending more than two hours watching television daily. A significantly lower proportion of children was found to have healthy eating habits (except regular exercise and fresh fruit consumption) and hygienic practices among families with unemployed fathers, single parents, being new arrivals, being recipients of CSSA and subsidies, with low monthly incomes (below HK$10,000), with parental education at primary level, and with relatives as the main caretakers (Tables 1 and 2).

**Table 1:** Association of socio-economic status with health behaviours of children.

	Family Monthly Income				Recipient of CSSA^#^			Father unemployed			Recipient of subsidy for school fees/transport		
Health behaviours	<$5,000 N = 276–328	$5,000–9,999 N = 1449–1611	≥$10,000N = 4037–4312	P	Yes N = 427–488	No N = 5725–6176	P	Yes N = 322–368	No N = 5494–5924	P	Yes N = 2698–2965	No N = 3454–3699	P
Spending at least 15 minutes quality time with their children everyday	70.7%	71.2%	74.0%	0.06	75.1%	73.2%	0.37	73.0%	73.3%	0.91	71.9%	74.5%	0.02*
Regular dental check up	5.8%	8.6%	13%	0.01*	9.1%	11.4%	0.15	9.9%	11.5%	0.38	9.1%	12.9%	0.01*
At least one serving of fruit daily	42.2%	42.1%	43.3%	0.66	41.1%	43.8%	0.24	34.9%	44.4%	0.01*	43.6%	43.6%	0.96
Having Breakfast daily	73.6%	76%	83.7%	0.01*	75.1%	81.9%	0.01*	76.4%	81.8%	0.01*	79.8%	82.7%	0.002*
Consumption of milk or dairy product at least twice a day	51.9%	55.6%	67.1%	0.01*	55.3%	64.2%	0.01*	56.2%	63.9%	0.003*	60.0%	66.3%	0.01*
Regular exercise	25.4%	22.8%	20%	0.01*	25.6%	21.1%	0.02*	23.5%	21.1%	0.29	22.0%	20.9%	0.31
Watching TV > 2hours daily	60.2%	62.1%	55.7%	0.01*	66.2%	56.3%	0.01*	66.2%	56.2%	0.01*	60.2%	54.5%	0.01*

Chi-square test of independence was used to analyze the differences of health behaviors of children amongst different socio-economic factors

^#^CSSA- Comprehensive Social Security Allowance

**Table 2:** Association of family background with health behaviours of children.

	Parental education				Main carer					New arrival to Hong Kong		
Health behaviours	Primary or below N = 290–340	Secondary N = 4280–4611	Post-secondary N = 1242–1286	P	Both parents N = 967–1044	Father N = 164–192	Mother N = 3679–3960	Relatives or others N = 1490–1605	P	Yes N = 1409–1558	No N = 4649–4958	P
Spending at least 15 minutes quality time with their children everyday	68.2%	72.5%	79.0%	0.01*	81.6%	72.3%	75.6%	62.6%	0.01*	73.9%	73.2%	0.62
Regular dental check up	5.9%	10.0%	17.6%	0.01*	14.3%	11.6%	10.3%	11.5%	0.007*	8.9%	12.1%	0.001*
At least one serving of fruit daily	39.5%	42.8%	47.7%	0.002*	43.9%	42.4%	43.5%	43.2%	0.98	47.1%	42.2%	0.01*
Having Breakfast daily	75.3%	80.2%	88.7%	0.01*	85.5%	71.9%	79.2%	85.5%	0.01*	79.9%	81.9%	0.08
Consumption of milk or dairy product at least twice a day	49.7%	63.4%	66.9%	0.01*	63.2%	55.0%	60.9%	69.7%	0.01*	53.3%	66.5%	0.01*
Regular exercise	23.9%	21.4%	21.6%	0.57	23.6%	20.8%	22.4%	17.0%	0.01*	27.2%	19.6%	0.01*
Watching TV > 2hours daily	63.2%	61.3%	39.7%	0.01*	52.5%	58.6%	56.0%	61.9%	0.01*	55.5%	57.3%	0.23

Chi square test of independence was used to analyse the difference between health behavours and socio-economic factors

Children were significantly less likely to have somatic complaints and anxiety/depression, as reflected by CBCL scores, if they came from families with higher incomes, families which are non-recipients of CSSA, families with father in employment, and families with higher parental education (not significant for anxious /depressed) (Table 3). The proportion of students with aggressive violent behaviors was found to be significantly lower among parents with secondary education or above (Table 3). Children with parental education at secondary level or post-secondary incurred higher ORs (OR 1.44 95% CI 1.2–1.75, OR 2.1 95% CI 1.59–2.8 respectively) of spending at least 15 minutes of close interactions with their children daily, whilst recipients of subsidies incurred lower OR (0.86; 95% CI 0.74–0.98).

**Table 3:** Correlation of CBCL scores with family and social background.

	Family Monthly Income				Parental Education				CSSA				Father Employment			
	Mean Score <$10,000	Mean Score ≥$10,000	Mean diff. (95% CI)	P	Mean Score Secondary or below	Mean Score Post-secondary or above	Mean diff. (95% CI)	P	Mean Score for recipient	Mean Score for non-CSSA	Mean diff. (95% CI)	P	Mean Score for un-employed	Mean Score for employed	Mean diff. (95% CI)	P
Anxious / Depressed	4.23	3.65	0.58	<0.001*	3.92	3.52	0.39	<0.001*	4.41	3.76	0.64	<0.001*	4.58	3.77	0.81	<0.001*
	N = 1469	N = 3741	(0.42–0.74)		N = 4172	N = 1186	(0.24–0.54)		N = 380	N = 5162	(0.35–0.94)		N = 277	N = 4992	(0.49–1.13)	
Somatic Complaints	4.11	3.40	0.72	<0.001*	3.81	3.08	0.73	<0.001*	4.19	3.56	0.64	0.001*	4.62	3.54	1.08	<0.001*
	N = 1412	N = 3649	(0.53–0.90)		N = 3958	N = 1155	(0.56–0.91)		N = 365	N = 5020	(0.26–1.01)		N = 265	N = 4853	(0.64–1.52)	
Aggressive Behaviours	11.67	11.01	0.66	0.003*	11.52	10.26	1.26	<0.001*	11.59	11.12	0.47	0.22	12.01	11.12	0.89	0.05
	N = 1317	N = 3453	(0.23–1.09)		N = 3747	N = 1081	(0.86–1.67)		N = 340	N = 4744	(-0.27–1.21)		N = 231	N = 4588	(-0.0006–1.78)	

T Test was utilized to analyse the difference in CBCL (Child Behaviour Check Lists) scores under different domain among different socio-economic groups

Tables 4 to 6 report the results of multivariate analyses performed to identify the significant independent variable(s) reflecting socio-economic status correlated with various health behaviors or health status.

**Table 4:** Odds ratios and their 95% confidence intervals from stepwise logistic regression analysis with family and social background (main carer, parental education, family income, recipient of subsidy, new arrival, employment status, school sponsoring body, district income) as independent variables and oral health (annual dental check) and healthy eating habits (breakfast every day, consumption of dairy products, at least one serving of fruit daily) as dependent variables.

	OR	95% CI	P
**Oral health (annual dental check)**			
***Main carer(s)***			P<0.05*
Both parents	1	Reference	—
Father	1.3	0.67–2.44	0.47
Mother	0.88	0.70–1.12	0.31
Relatives or others	0.72	0.6–0.95	0.02*
***Family Income***			P <0.01*
<$5,000	1	Reference	—
$5,000–$9,999	1.28	0.67–2.4	0.47
$10,000–$19,999	1.38	0.73–2.47	0.35
$20,000–$29,999	1.80	0.96–3.38	0.07
$30,000 or above	2.6	1.38–4.76	<0.003*
**Eating breakfast everyday**			
***Parental education***			P<0.001**
Primary school or below	1	Reference	—
Secondary school	1.18	0.87–1.61	0.29
Post-secondary	1.91	1.32–2.78	<0.001**
***Main caretakers***			P<0.01*
Both parents	1	Reference	—
Father	0.53	0.33–0.84	0.007*
Mother	0.71	0.58–0.89	<0.002*
Relatives or others	0.93	0.72–1.20	0.55
*Family monthly income*			<0.01*
<$5,000	1	Reference	—
$5,000–$9,999	1.06	0.74–1.52	0.74
$10,000–$19,999	1.47	1.03–2.09	0.03*
$20,000–$29,999	1.8	1.22–2.67	0.03*
$30,000 or above	1.63	1.1–2.39	0.01*
**Consumption of dairy products at least twice daily**	P<0.01* P<0.001**		
***Family Income***			
<$5,000	1	Reference	—
$5,000–$9,999	1.17	0.86–1.60	0.33
$10,000–$19,999	1.59	1.17–2.16	<0.003*
$20,000–$29,999	1.62	1.16–2.25	<0.005*
$30,000 or above	1.77	1.27–2.47	<0.01*
***New arrival to HK***			
Ordinary resident	1	Reference	—
New arrival	0.65	0.56–0.75	<0.001**
***Employment status***			
Mothers employed	1	Reference	—
Mothers unemployed	0.44	0.26–0.74	0.002*
**Consumption of at least 1 serving of fruit**			
***Parental education***			
Primary education or below	1	Reference	—
Secondary education	1.27	1.17–2/11	0.003*
Post-secondary	1.57	1.17–2.11	<0.003*
***New arrival to Hong Kong***			
Ordinary resident	1	Reference	—
New arrival	1.35	1.17–1.55	<0.001**
***Employment status***			
Father employed	1	Reference	—
Father unemployed	0.63	0.48–0.84	0.001*
***School located in districts where median household income***			
$20,000 or above	1	Reference	
$16,000-$19,500	0.84	0.72–0.98	0.03*
$15,00 or below	0.88	0.77–1.01	0.07

**Table 5:** Odds ratios and their 95% confidence intervals from stepwise logistic regression analysis with family and social background (main carer, parental education, family income, recipient of subsidy, new arrival, employment status, school sponsoring body, district income) as independent variables and physical activity (30 minutes of moderate and vigorous exercise five times a week or above) and sedentary lifestyle (over 2 hour of TV viewing daily) as dependent variable.

	OR	95% CI	P
**Over last week, 30 minutes or above moderate or vigorous exercise for at least 5 days**			P<0.01* P<0.001**
***Main caretakers***			
Both parents	1	Reference	—
Father	0.46	0.75–1.87	0.46
Mother	0.95	0.79–1.14	0.55
Relatives or others	0.73	0.58–0.90	0.004*
***New arrival to HK***			
Ordinary resident	1	Reference	—
New arrival	1.35	1.15–1.58	<0.001**
**Over last one week, average TV viewing time over 2 hours everyday**			
***Parental education***			
Primary education or below	1	Reference	—
Secondary education	0.82	0.62–1.08	0.16
Post-secondary	0.33	0.24–0.45	<0.001**
***Main carer***			<0.001**
Both parents	1	Reference	—
Father	0.81	0.54–1.21	0.31
Mother	1.1	0.94–1.30	0.21
Relatives or others	1.52	1.27–1.83	0.001**
***New Arrival to HK***			
Local resident	1		
New Arrival	0.87	0.75–1.00	0.05*
***Father’s employment status***			
Working	1		
Unemployed	1.46	1.1–1.9	0.01*
**School located in districts where median household income**			
$20,000 or above	1		
$16,000-$19,500	1.01	0.86–1.12	0.88
$15,000 or below	1.17	1.02–1.34	0.02

**Table 6:** Odds ratios and their 95% confidence intervals from stepwise logistic regression analysis with family and social background (main carer, parental education, family income, recipient of subsidy, new arrival, employment status, school sponsoring body, district income) as independent variables and exposure to passive smoking as dependent variables.

	OR	95% CI	P
**Over last one month, child has exposed to passive smoking**			P<0.01* P<0.001**
***Parental education***			
Primary education or below	1	Reference	—
Secondary education	1.14	0.82–1.57	0.44
Post-secondary	0.68	0.48–0.97	<0.03*
***Family monthly income***			
<$5,000	1	Reference	—
$5,000–$9,999	0.74	0.49–1.10	0.14
$10,000–$19,999	0.72	0.5–1.09	0.12
$20,000–$29,999	0.59	0.39–0.91	0.02*
$30,000 or above	0.52	0.34–0.79	0.002*
***New arrival to HK***			
Ordinary resident	1	Reference	—
New arrival	0.84	0.71–0.99	0.04*
CSSA^#^			
Non-recipient	1		
Recipient	1.6	1.09–2.37	0.02*
**School located in districts where median household income**			
$20,000 or above	1	Reference	
$16,000-$19,500	1.11	0.93–1.31	0.25
$15,000 or below	1.24	1.06–1.44	0.006*

^#^ CSSA (Comprehensive Social Security Allowance)

### Oral health and healthy eating habits

Children with relatives as main caregivers were less likely to have annual visit to the dentist (Table 4). Children with family income HK$30,000 or above and parental education at secondary and post-secondary incurred higher ORs associated with the habit of eating breakfast, and lower OR was observed among children with only one parent as main caregiver (Table 4).

Children with family income above HK$10,000 incurred higher OR of consumption of dairy product at least twice daily, whilst lower OR was seen for new arrivals and unemployed mothers and also students attending kindergartens in districts with median household income below $20,000 (Table 4). Children with parental education at secondary level or above and the new arrivals incurred higher ORs for the consumption of at least one serving of fresh fruits daily, whilst children with unemployed fathers incurred lower OR (Table 4).

### Physical activities

Children with relatives as main caretakers incurred lower OR of regular exercises whilst the new arrivals bore higher OR (Table 5). Fathers unemployed, relatives as main caretakers and students located in districts with median household income below HK$20,000 incurred higher OR for watching television over two hours daily, whilst children with parental education at secondary level or above incurred lower OR (Table 5) Exposure to passive smoking.

A lower OR was observed for children with parental education at post-secondary level, mother as main carer and family income above HK$20,000 with regard to exposure to passive smoking (Table 6). Children located in districts with median household income HK$20,000 and below, recipient of CSSA and new arrival incurred lower OR with regard to exposure to passive smoking.

## Discussion

As health related behaviors of kindergarten children including emotional health were found to be significantly correlated with socio-economic factors of the families in the affluent region of Hong Kong, our null hypothesis was refuted.

Hatt and Waters studying respiratory illnesses and diarrhea highlighted the significance of parental education and employment as well as resources within the family on child health including emotional health and healthy lifestyle [8]. Kawachi *et al* have identified several pathways of the effect of income distribution on health [34]. One pathway is reduced access to life opportunities, material resources, and opportunity structures. This would explain the lower likelihood of adopting healthy behaviours by children from families of low income, low parental education, new arrival, unemployed father, recipients of CSSA and subsidies, and living in districts with lower median household income. Subjective social status was found to be associated with mental health and self-rated health [16], with the perceived victimization being particularly relevant for the low income minority population. This might mediate the effect of subjective social status on mental health. Families with limited resources might not offer psycho-social resources for cognitive and emotional support to raise resilient children [35]. This would explain the higher likelihood of aggressive behaviors among children with low parental education and somatic complaints among those with low family income and low parental education (Table 4).

The quality and quantity of parent-child social interaction have been shown to influence socio-emotional developments [36]. In eastern cultures, parents put less emphasis on the psycho-social aspects of development and the importance of environment and socio-cultural influences on development. Manifestations as physical diagnostic labels are more acceptable especially for those in lower socio-economic groups. We accordingly observed higher prevalence of somatic complaints in these groups.

Marmot *et al* analyzed factors likely to close the health gaps [3]. Some of those factors, such as universal health care, social protection throughout life, fair employment and decent work, health equity in all policies and systems, fair financing, market responsibility, and political empowerment, warrant further development and actualization in Hong Kong. A robust and cost-effective health care system itself is a social determinant of health, both influenced by and influencing the effect of other social determinants [3]. Most of the well developed countries adopt the principle of universal coverage so that everyone within the country could access services according to his/her needs including preventive health care. An effective primary care system would improve population health and reduce health inequities as it serves health continuum covering acute and chronic care as well as preventive care [37].

Hong Kong operates a dual health care system with private sector providing 70% of primary medical care and public sector providing 90% of hospital care. Delivery of primary health care in Hong Kong is therefore fragmented and less efficient without universal coverage. The high level of inappropriate utilization of health services, e.g., emergency services [38], and the lack of co-ordination between primary and secondary care during the SARS outbreak [39] reflected problem of efficiency and integration. A system of good-quality primary care services might be able to reduce some of the ultimate consequences of social inequalities at the population level by lowering aggregate levels of risk factors (such as hypertension, smoking, weight gain), improving wide screening and early diagnosis activities, and developing system of care coordination [40]. The future health care reform in Hong Kong should seriously consider universal access to primary health care.

Hong Kong is a highly economic driven society encouraging free trade and free market. The low taxation and market driven economy do not place strong emphasis on fair financing, fair employment and decent work, and market responsibility. One example is the pursuit for minimum wage. The concerned groups had advocated it for years before it was finally implemented in 2011. In this study, no statistical significant differences were observed for various types of health outcomes whether the kindergartens were operated by major School Sponsoring Bodies or not after adjustment of all other variables. The attention should then be focusing on improving living standard at individual family level.

The emerging countries would have exposed their population to high risk during the rapid process of economic development and urbanization over the last few decades without comprehensive mitigating measures in place [41, 42]. As the world encounters new economic, social and environmental challenges, the improvements in health and wealth should be equally distributed and sustainable [43]. Apart from socio-economic status of their own families, the median household income of the districts were also found to be an independent factors related to exposure to passive smoking, consumption of fresh fruits daily and excess time spent on television in this study. The improvement of standard living should aim to benefit the wider community rather than just individual families. Smaller income differences, more trusts of one another, and greater participation in communal activities would lead to lower overall mortality rates [44], better self-rated health [45], and less psychological distress [18]. Barten *et al* argued for long term multi-sectorial approaches to address the social determinants of health in urban settings and adopted a multiple levels approach tackling issues of governance, the politics of power, decision making, and empowerment [46]. Tackling health inequities is a political imperative which requires leadership, political courage, social action, and a sound evidence-based and progressive public policy [47]. Limitations of study.

This study incurs several limitations. Firstly, the respondents were selected in a non-randomized manner. However, the study population had included students from territory wide with a large sample size, giving a lower error rate (a sample of 4,000 gives an error ±3%). There were differences in level of education, unemployment rates, occupations and monthly household income between respondents and Hong Kong population. Our respondents had higher education level and higher proportion working as professional and associated professionals but higher unemployment rate and lower proportion with income above HK$20,000. The key socio-demographic characteristics of the study population are not markedly skewed towards one direction in comparison with Hong Kong population (S1 Appendix). The association in general population would be similar to current study.

Secondly, the questionnaire does not contain all the vital information related to health behaviors, health status, and emotional health. There are still several potential confounding factors to be explored, such as cultural beliefs and lifestyles of parents. The numbers and availability of dentists in the neighbourhood might also have impact on oral health. However, inclusion of these and other confounding factors would render the questionnaires too long and the study processes not streamlined. Parent-completed questionnaires would have measurement errors with the possibility of higher-income parents tended to report better outcomes. The parent-feeding style questionnaire evolved from the dietary component of this questionnaire has been shown to have high reliability and validity by a recent study [48]. Although parent-completed questionnaire has potential bias, the reliability and validity is still reasonable in our local context. As the prevalence of passive smoking was not high, we did not conduct further analysis across different socio-economic groups. As primary caregivers might not fully comprehend smoking hygiene of other family members, the questionnaire did not include that aspect.

Lastly, a cross sectional study could not define causality and direction of associations with a high degree of certainty. Reverse causation would also be a concern. A longitudinal study would be planned to establish the causal link. Re-analyses of past, present, and future data would allow application of Hill’s criteria [49] (strength, consistency, specificity, temporality, biological gradient, plausibility, coherence, experimental evidence, and analogy) to establish casual relationships so that the impact of interventions can be assessed.

## Conclusion

The null hypothesis was not supported. A strong gradient was still found to exist among different socio-economic groups for various health-related behaviors in the affluent region of Hong Kong. If this trend continues, this would lead to future inequalities in adult health and policy action should be target to close the gap rather than boosting economic development at macro-level.

## Supporting Information

S1 Appendix The Characteristics of the Sampling population comparing to the Population of Hong Kong. The socio-demogrpahy characteristics of the sampling population in comparison to Hong Kong population have included household income, household size, parental education level, occupation and employment status. Other characteristics such as numbers of kindergartens in Hong Kong as well as total numbers of preschool students in K1 and K2 have also been included for comparison.
(DOC)

S2 Appendix Prevalence of various health and hygiene behaviours of children. The hygiene behaviours included hand washing and oral hygiene. Various health behaviours included dietary habits of consumption of fresh fruit and vegetables, water and diary product consumption as well as consumption of high calorie drinks. Level of physical activities and sedentary lifestyle reflected by time spent on television were also included.
(DOC)
