# Supplementary material for: Health Disparity Still Exists in an Economically Well-Developed Society in Asia
Source: PLoS One. 2015 Jun 22;10(6):e0130424. doi: 10.1371/journal.pone.0130424 (PMC4476700; doi:10.1371/journal.pone.0130424)
Supplement: S1 Appendix — The socio-demogrpahy characteristics of the sampling population in comparison to Hong Kong population have included household income, household size, parental education level, occupation and employment status. Other characteristics such as numbers of kindergartens in Hong Kong as well as total numbers of preschool students in K1 and K2 have also been included for comparison. (DOC) [file pone.0130424.s001.doc]

**Appendix 1: The Characteristics of the Sampling population comparing to the Population of Hong Kong.**

| Variables | Respondents (N) | Hong Kong Population |
| --- | --- | --- |
| Number of kindergartens | 78 | 1062 ^1^ |
| Number (K1 and K2) of pre-schools children | 7057 | 94274 ^1^ |
| Average domestic household size | 4.1 | 3.1 ^1^ |
|  |  |  |
| Levels of parental education |  |  |
| Primary school level or below^#^ | 5.5% | 25.4% ^2^ |
| Secondary school level^#^ | 73.9% | 51.6%^2^ |
| Tertiary or above | 20.6% | 23%^2^ |
| Unemployment rate | 7.8% | 4.8% ^3^ |
| Occupation distribution: |  |  |
| Professionals and associate professionals, managers | 49% | 33%^2^ |
| Clerks, services and sales workers | 42% | 33.3%^2^ |
| Skilled occupation | 4% | 14.7%^2^ |
| Unskilled occupation | 5% | 19.1%^2^ |
| Average household monthly income * |  |  |
| $9999 or below | 31.1% | 27.9%^2^ |
| $10,000 - $19, 999 | 32.9% | 27.9%^2^ |
| $20,000 - $29,999 | 14.1% | 17.4% ^2^ |
| $30, 000 or above | 22.0% | 26.8%^2^ |

Education and Manpower Bureau, <http://www.emb.gov.hk/index.aspx?langno=1&nodeid=1037> (download on 18/8/2006)

The Census and Statistics Department (Hong Kong), 2006

Hong Kong Yearbook 2006 www.yearbook.gov.hk/2006/en/fact

*Median monthly income from main employment of Hong Kong was 10,000 in 2006

# Primary education six years, secondary education 7 years

# 
