# Supplementary material for: Health Disparity Still Exists in an Economically Well-Developed Society in Asia
Source: PLoS One. 2015 Jun 22;10(6):e0130424. doi: 10.1371/journal.pone.0130424 (PMC4476700; doi:10.1371/journal.pone.0130424)
Supplement: S2 Appendix — The hygiene behaviours included hand washing and oral hygiene. Various health behaviours included dietary habits of consumption of fresh fruit and vegetables, water and diary product consumption as well as consumption of high calorie drinks. Level of physical activities and sedentary lifestyle reflected by time spent on television were also included. (DOC) [file pone.0130424.s002.doc]

# Appendix 2: Prevalence of various health and hygiene behaviours of children ( ) denote the denominator

|  | ***Percentage (%)*** |
| --- | --- |
| ***Hygienic practices*** | |
| Wash hands before meals | 96.4% *(6619)* |
| Wash hands automatically without reminding or supervision by adults after using toilet | 52% *(4,000)* |
| Wash hands with soap/liquid soap | 95.7% *(6493)* |
| Brush teeth after getting up | 93.1% *(6285)* |
| Brush teeth before going to bed | 86.4% *(5868)* |
| Brush teeth after each meal | 32.0% *(2106)* |
| Annual dental check up | 11.2% *(715)* |
| ***Dietary Habits*** | |
| Have breakfast everyday | 81.3% *(5635)* |
| Consume at least 1 bowls of cooked vegetables or melons on average per day | 33.9% *(2347)* |
| Consume at least 1 portion of fresh fruit on average per day | 43.5% *(3014)* |
| Consume milk or dairy products at least 2 times on average per day | 63.2% *(4379)* |
| Consume fizzy or high sugar content drinks less than 4 times per week | 88.5% *(6134)* |
| Drink at least 5 glasses of water everyday | 16.2% *(1122)* |
| ***Exercise*** | |
| Participate in physical activity of moderate intensity or above at least 60 minutes each time for 1 days or above in a week. | 16.5% *(6420)* |
| Watch TV for less than 2 hours on average per day | 43.0% *(2919)* |
